# Staphylococcal scalded skin syndrome in a premature newborn caused by methicillin-resistant Staphylococcus aureus: case report: Síndrome da pele escaldada estafilocócica em um recém-nascido prematuro causada por Staphylococcus aureus meticilina-resistente: relato de caso

**DOI:** 10.1590/1516-3180.2013.79400715

**Published:** 2015-03-17

**Authors:** Andreas Hörner, Rosmari Hörner, Adenilde Salla, Melise Silveira Nunes, Litiérri Razia Garzon, Roberta Filipini Rampelotto, Rosiéli Martini, Silvana Oliveira dos Santos, Lívia Gindri, Mônica de Abreu Rodrigues, Cláudia Giacomolli

**Affiliations:** I Undergraduate Student, Universidade Federal de Santa Maria (UFSM), Santa Maria, Rio Grande do Sul, Brazil; II PhD. Associate Professor, Department of Clinical and Toxicological Analyses, Universidade Federal de Santa Maria (UFSM), Santa Maria, Rio Grande do Sul, Brazil; III BSc. Pharmacist, Bacteriology Laboratory, Hospital Universitário de Santa Maria (HUSM), Universidade Federal de Santa Maria (UFSM), Santa Maria, Rio Grande do Sul, Brazil; IV BSc. Master's Student, Postgraduate Pharmaceutical Sciences Program, Universidade Federal de Santa Maria (UFSM), Santa Maria, Rio Grande do Sul, Brazil; V MSc. Doctoral Student, Postgraduate Pharmaceutical Sciences Program, Universidade Federal de Santa Maria (UFSM), Santa Maria, Rio Grande do Sul, Brazil; VI MD. Pharmacist, Universidade Federal de Santa Maria (UFSM), Santa Maria, Rio Grande do Sul, Brazil

**Keywords:** Staphylococcal scalded skin syndrome, Methicillin-resistant Staphylococcus aureus, Infant, newborn, Infant, premature, Impetigo, Síndrome da pele escaldada estafilocócica, Staphylococcus aureus resistente à meticilina, Recém-nascido, Prematuro, Impetigo

## Abstract

**CONTEXT:**

Staphylococcal scalded skin syndrome is an exfoliative skin disease. Reports of this syndrome in newborns caused by methicillin-resistant *Staphylococcus aureus* are rare but, when present, rapid diagnosis and treatment is required in order to decrease morbidity and mortality.

**CASE REPORT:**

A premature newly born girl weighing 1,520 g, born with a gestational age of 29 weeks and 4 days, developed staphylococcal scalded skin syndrome on the fifth day of life. Cultures on blood samples collected on the first and fourth days were negative, but *Pseudomonas aeruginosa* and *Enterococcus* sp. (vancomycin-sensitive) developed in blood cultures performed on the day of death (seventh day), and *Pseudomonas aeruginosa* and *Serratia marcescens* were identified in cultures on nasopharyngeal, buttock and abdominal secretions. In addition to these two Gram-negative bacilli, methicillin-resistant *Staphylococcus aureus* was isolated in a culture on the umbilical stump (seventh day). The diagnosis of staphylococcal scalded skin syndrome was based on clinical criteria.

## Abstract (pt)

**CONTEXTO:**

A síndrome da pele escaldada estafilocócica é uma doença esfoliativa de pele. São raros os relatos desta síndrome causada por *Staphylococcus aureus* resistente à meticilina em neonatos, mas, quando presentes, exigem diagnóstico e tratamento rápidos para diminuir a morbidade e mortalidade.

**RELATO DE CASO:**

Uma menina recém-nascida prematura, pesando 1.520 g ao nascimento, com idade gestacional de 29 semanas e 4 dias, desenvolveu síndrome da pele escaldada estafilocócica no quinto dia de vida. As culturas de sangue coletadas no primeiro e quarto dias foram negativas, mas houve desenvolvimento de *Pseudomonas aeruginosa e Enterococcus* sp. (vancomicina sensível) na hemocultura realizada no dia do óbito (sétimo dia) e *Pseudomonas aeruginosa e Serratia marcescens* foram identificadas nas culturas de secreção da nasofaringe, nádega e da secreção abdominal. Na cultura do coto umbilical (sétimo dia), além desses dois bacilos Gram-negativos, foi isolado o *Staphylococcus aureus* resistente à meticilina. O diagnóstico da síndrome da pele escaldada estafilocócica foi baseado em critério clínico.

## INTRODUCTION

Staphylococcal scalded skin syndrome is a rapidly expanding exfoliative disease of the skin and is characterized by blistering of the skin.^1^ It is also known as Ritter's disease and usually affects newborn between the first three and the 16th days of life. It is extremely rare in low-weight newborns.^1^ This syndrome can present as a severe and fatal disease in extremely premature newborns. 2 It presents numerous skin complications and the severity of the lesions may range from localized bullous lesions (bullous impetigo) to severe exfoliation lesions, generally covering smaller portions of the arms and trunk3 but possibly extending to most of the patient's body surface.^4^^-^ 6

In this study, we present a case of staphylococcal scalded skin syndrome in a premature newborn caused by methicillin-resistant *Staphylococcus aureus*, with a fatal outcome due to sepsis caused by *Pseudomonas aeruginosa*, *Enterococcus* sp. and *Serratia marcescens*, which was diagnosed at the University Hospital of Santa Maria (Hospital Universitário de Santa Maria, HUSM), Santa Maria, Rio Grande do Sul, Brazil.

## CASE REPORT

The patient was a premature newly born girl weighing 1,520 g at birth, who was born in March 2010 by cesarean section, with a length of 38 cm and gestational age of 29 weeks and 4 days. The pregnancy was uncomplicated and the mother (19 years of age) had made three prenatal care visits and had received corticosteroids before childbirth. The Apgar scores were 4 and 9 in the first and fifth minutes after birth, respectively. Rupture of the newborn's membranes occurred at childbirth, and the infant was immediately transferred to the neonatal intensive care unit. In this unit, a peripherally inserted central catheter was implanted in the left basilic vein, with the purposes of hydration, parenteral nutrition and medication.

Starting on the second day, the newborn underwent ocular hygiene procedures using 0.9% saline and respiratory therapy. On the fifth day, because of large amounts of discharge from the eyes, use of gentamicin eye drops was started, along with simple phototherapy.

Cultures on blood samples collected on the first and fourth days after birth presented negative results. C-reactive protein assays, also performed on the first and fourth days, showed results of 0.01 mg/dl and 0.28 mg/dl respectively (reference values: less than 0.9 mg/dl). Whole blood counts performed on the same days showed leukocyte count of 12,400/mm^3^ (3% rod neutrophils, 26.4% segmented neutrophils and 59.9% lymphocytes), hematocrit of 44.7%, hemoglobin of 14.6 g/dl and platelet count of 146,000/mm^3^ on the first day; and leukocyte count of 7,800/mm^3^ (5% rod neutrophils, 38% segmented neutrophils and 46% lymphocytes), hematocrit of 42.3%, hemoglobin of 13.5 g/dl and platelet count of 131,000/mm^3^ on the fourth day.

On the fifth day of life, erythematous bullous lesions were observed in the nasal area, right index finger area and abdominal area. Antibiotic therapy was started, consisting of oxacillin, under the diagnostic hypothesis of staphylococcal scalded skin syndrome. On the next day, these lesions progressed to bleeding with yellowish discharge. Use of oxacillin was suspended and administration of clindamycin, morphine and dipyrone was started. 

Exfoliative lesions occupied approximately 30% of the baby's body surface on the seventh day: thus, oxacillin and cefepime were administered concomitantly. Blood, nasopharyngeal secretion, buttock secretion, abdominal secretion and umbilical stump samples were collected. Nasopharyngeal, buttock and abdominal secretion cultures showed the presence of *Pseudomonas aeruginosa* and *Serratia marcescens*. In addition to these two bacteria, methicillin-resistant *Staphylococcus aureus* was also identified in the umbilical stump. From this blood culture, *Pseudomonas aeruginosa *and *Enterococcus* sp. were isolated. At the end of this day (seventh day), the newborn evolved to death because of severe sepsis. 

Table 1 shows the sensitivity profile of the antimicrobial agents acting on the samples from the newborn. These antimicrobial susceptibility tests were conducted in accordance with the recommendations of the Clinical and Laboratory Standards Institute (CLSI) of the year of this study.^7^

**Table 1.** Sensitivity profile of antimicrobials acting on samples from the newborn

Sample	Microorganisms	Antimicrobial agents																															
		AMI	AMC	AMP	AMS	AZM	ATM	CPM	CAZ	CRO	CIP	CLI	STR	GEN	IPM	LEV	LNZ	NIT	MEM	OXA	PEN	PTZ	SXT	TEI	TOB	VAN	CFZ	CTX	CFX	CFL	TIM	ERY	RIF
Blood	1	S	*	*	*	*	S	S	S	R	S	*	*	S	S	S	*	*	*	*	*	S	*	*	*	*	*	*	*	*	*	*	*
	2	*	*	S	*	R	*	*	*	R	S	R	S	*	*	S	S	S	*	R	S	*	*	S	*	S	*	*	*	*	*	*	*
Swab umbilical stump	1	S	*	*	*	*	R	S	R	R	S	*	*	S	S	S	*	*	*	*	*	R	*	*	S	*	*	I	*	*	R	*	*
	3	S	R	R	R	*	R	S	R	R	S	*	*	S	S	S	*	*	*	*	*	R	R	*	I	*	R	R	R	R	R	*	*
	4	*	R	R	R	*	*	*	*	R	*	R	*	R	R	S	S	*	R	R	R	*	S	*	*	S	R	*	*	*	*	I	R
Swab buttock secretion	1	S	*	*	*	*	R	S	R	R	S	*	*	S	S	S	*	*	*	*	*	R	*	*	S	*	*	I	*	*	R	*	*
	3	S	R	R	R	*	R	S	R	R	S	*	*	S	S	S	*	*	*	*	*	R	R	*	I	*	R	R	R	R	R	*	*
Swab abdominal secretion	1	S	*	*	*	*	R	S	R	R	S	*	*	S	S	S	*	*	*	*	*	R	*	*	S	*	*	I	*	*	R	*	*
	3	S	R	R	I	*	R	S	R	R	S	*	*	S	S	S	*	*	*	*	*	R	R	*	I	*	R	R	R	R	R	*	*
Swab nasopharyngeal secretion	1	S	*	*	*	*	R	S	R	R	S	*	*	S	S	S	*	*	*	*	*	R	*	*	S	*	*	I	*	*	R	*	*
	3	S	R	R	I	*	R	S	R	R	S	*	*	S	S	S	*	*	*	*	*	R	R	*	I	*	R	R	R	R	R	*	*

Microorganisms: 1= Pseudomonas aeruginosa; 2 = Enterococcus sp.; 3 = Serratia marcescens; 4 = Staphylococcus aureus. Antimicrobial agent abbreviations: AMI = amikacin; AMC = amoxicillin/clavulanic acid; AMP = ampicillin; AMS = ampicillin/sulbactam; AZM = azithromycin; ATM = aztreonam; CPM = cefepime; CAZ = ceftazidime; CRO = ceftriaxone; CIP = ciprofloxacin; CLI = clindamycin; STR = streptomycin; GEN = gentamicin; IPM = imipenem; LEV = levofloxacin; LNZ = linezolid; NIT = nitrofurantoin; MEM = meropenem; OXA = oxacillin; PEN = penicillin; PTZ = piperacillin/tazobactam; SXT = trimethoprim/sulfamethoxazole; TEI = teicoplanin; TOB = tobramycin; VAN = vancomycin; CFZ = cefazolin; CTX = cefotaxime; CFX = cefuroxime; CFL = cephalexin; TIM = ticarcillin/clavulanic acid; ERY = erythromycin; RIF = rifampin. Sensitivity profile: S = sensitive; I = intermediate; R = resistant; * = not tested.

## DISCUSSION

The skin is usually inhabited by resident bacteria, which constitute the microbiota, and transient bacteria, which occasionally colonize it.^8^ Bacterial infections of the skin may represent a primary cutaneous pathogenic process or a secondary cutaneous manifestation that started in another organ. They may be suppurative, because of the proliferation of bacteria or because of hypersensitivity to bacterial antigens.^3^

Staphylococcal scalded skin syndrome caused by methicillin-resistant *Staphylococcus aureus* or oxacillin-resistant *Staphylococcus aureus* is characterized as a severe infection in neonates, although it is extremely rare in low-weight newborns.^9^ From reviewing the literature, using the Medline (http://www.ncbi.nlm.nih.gov/pubmed/), Embase ((http://www.elsevier.com/online-tools/embase), SciELO (http://www.scielo.org/php/index.php) and Lilacs (http://lilacs.bvsalud.org/en/) databases, we found one article^9^ describing staphylococcal scalded skin syndrome caused by methicillin-resistant *Staphylococcus aureus *(Table 2). 

**Table 2.** Review of medical databases using the descriptors corresponding to the main features presented by the patient, conducted on December 9, 2013

Database	Search strategy	Results
PubMed	(“Staphylococcal scalded skin syndrome” [MeSH]) AND “Methicillin-resistant Staphylococcus aureus” [MeSH]) AND “Infant, Premature”[MeSH] OR (“Newborn”[MeSH])	1 article
Embase	(“Staphylococcal scalded skin syndrome” [MeSH]) AND “Methicillin-resistant Staphylococcus aureus” [MeSH]) AND “Infant, Premature”[MeSH] OR (“Newborn”[MeSH])	0 article
SciELO	(“Staphylococcal scalded skin syndrome” [MeSH]) AND “Methicillin-resistant Staphylococcus aureus” [MeSH]) AND “Infant, Premature”[MeSH] OR (“Newborn”[MeSH])	0 article
Lilacs	(“Staphylococcal scalded skin syndrome” [MeSH]) AND “Methicillin-resistant Staphylococcus aureus” [MeSH]) AND “Infant, Premature”[MeSH] OR (“Newborn”[MeSH])	0 article

*Staphylococcus aureus* produces exfoliative toxins, which are responsible for the extensive erythematous skin peeling that is characteristic of staphylococcal scalded skin syndrome.^9^ It has been suggested that the action of these toxins is due to their ability to cleave: thus, a protease that targets desmoglein desmosomal adhesion molecule-1^10^ leads to loss of cell-to-cell contact in the epidermis,^11^ thereby causing the formation of large bubbles that are fragile and burst easily, which leaves this area of skin wet and unprotected, such that development of this syndrome is favored.^6^

In neonates, staphylococcal scalded skin syndrome produces strains that colonize sites such as the nose and umbilical cord.^12^ Neonates are also exposed to the risk of acquiring infections caused by *Staphylococcus aureus*, which colonizes both mothers and staff in the hospital environment,^13^^,^ 14 considering that one third of the immunocompetent population carries this microorganism.^6^

The resistance presented by methicillin-resistant *Staphylococcus aureus* extends to all β-lactam antibiotics and therefore this is an etiological agent of utmost importance, with regard to both nosocomial infections (hospital-acquired methicillin-resistant *Staphylococcus aureus*) and community-acquired infections. Patients with methicillin-resistant *Staphylococcus aureus* present a high mortality rate.^15^

The treatment for infections caused by methicillin-resistant *Staphylococcus aureus* has become increasingly complicated because of their increasing resistance to antimicrobial agents and diversity of strains. Therefore, these treatments are based on personalized therapies.^16^

Fatal outcomes are rare in children, but the mortality rate exceeds 50% among adult patients with severe underlying diseases.^17^ When death occurs among newborns and children, it usually results from secondary bacterial infection of skin areas devoid of skin.^17^

The most likely explanation for staphylococcal scalded skin syndrome affecting children under five years of age and newborns is that these individuals lack immunity to exfoliative toxins and also present renal immaturity, which results in decreased clearance of these toxins.^15^

## CONCLUSION

We emphasize the importance of maintaining suspicion, making a prompt diagnosis of this syndrome and instituting early treatment, which may inhibit secondary bacterial infections and consequently decrease mortality among newborns. Isolation of the microorganism in biological materials makes it possible to ascertain the resistance profile, which considerably improves the prognosis. To our knowledge, this was the first episode of staphylococcal scalded skin syndrome caused by methicillin-resistant Staphylococcus aureus that occurred in our institution.
